# Revealing the Pharmacological Mechanism of *Acorus tatarinowii* in the Treatment of Ischemic Stroke Based on Network Pharmacology

**DOI:** 10.1155/2020/3236768

**Published:** 2020-10-31

**Authors:** FengZhi Liu, Qian Zhao, Suxian Liu, Yingzhi Xu, Dongrui Zhou, Ying Gao, Lingqun Zhu

**Affiliations:** ^1^Key Laboratory of Chinese Internal Medicine of Ministry of Education and Beijing, Dongzhimen Hospital, Beijing University of Chinese Medicine, Beijing 100700, China; ^2^Beijing University of Chinese Medicine, Beijing 100029, China; ^3^Pharmaceutical Science, Beijing University Health Science Center, Beijing 100089, China; ^4^Longhua Hospital Shanghai University of Traditional Chinese Medicine, Shanghai 200032, China; ^5^Key Office of Encephalopathy TCM Research, Dongzhimen Hospital, Beijing University of Chinese Medicine, Beijing 100700, China

## Abstract

**Aim:**

Stroke is the second significant cause for death, with ischemic stroke (IS) being the main type threatening human being's health. *Acorus tatarinowii* (AT) is widely used in the treatment of Alzheimer disease, epilepsy, depression, and stroke, which leads to disorders of consciousness disease. However, the systemic mechanism of AT treating IS is unexplicit. This article is supposed to explain why AT has an effect on the treatment of IS in a comprehensive and systematic way by network pharmacology.

**Methods and Materials:**

ADME (absorbed, distributed, metabolized, and excreted) is an important property for screening-related compounds in AT, which were screening out of TCMSP, TCMID, Chemistry Database, and literature from CNKI. Then, these targets related to screened compounds were predicted via Swiss Targets, when AT-related targets database was established. The gene targets related to IS were collected from DisGeNET and GeneCards. IS-AT is a common protein interactive network established by STRING Database. Gene Ontology (GO) and Kyoto Encyclopedia of Genes and Genomes (KEGG) pathway enrichment were analysed by IS-AT common target genes. Cytoscape software was used to establish a visualized network for active compounds-core targets and core target proteins-proteins interactive network. Furthermore, we drew a signal pathway picture about its effect to reveal the basic mechanism of AT against IS systematically.

**Results:**

There were 53 active compounds screened from AT, inferring the main therapeutic substances as follows: bisasaricin, 3-cyclohexene-1-methanol-*α*,*α*,4-trimethyl,acetate, *cis*,*cis*,*cis*-7,10,13-hexadecatrienal, hydroxyacoronene, nerolidol, galgravin, veraguensin, 2′-o-methyl isoliquiritigenin, gamma-asarone, and alpha-asarone. We obtained 398 related targets, 63 of which were the same as the IS-related genes from targets prediction. Except for GRM2, remaining 62 target genes have an interactive relation, respectively. The top 10 degree core target genes were IL6, TNF, IL1B, TLR4, NOS3, MAPK1, PTGS2, VEGFA, JUN, and MMP9. There were more than 20 terms of biological process, 7 terms of cellular components, and 14 terms of molecular function through GO enrichment analysis and 13 terms of signal pathway from KEGG enrichment analysis based on *P* < 0.05.

**Conclusion:**

AT had a therapeutic effect for ischemic via multicomponent, multitarget, and multisignal pathway, which provided a novel research aspect for AT against IS.

## 1. Introduction

13.7 million people suffered from stroke every year in the world, the second largest reason causing death with around 5.5 million dead [1, 2]. It not only killed and enabled the elderly people [3] but also made a negative effect for the youth that morbidity rate and incidence had enhanced among them. Especially, mortality rate among young people suffering from stroke in developing countries was in a uptendency, which highly increased disease burden [4]. Over past 30 years, the same situation happened in China [5], where ischemic stroke is the major type of stroke, accounting for 70.8% [6]. The ratio of mortality rate to disablility rate among the youth in China was 34.5% to 37.1% [7, 8]. Thrombolytic therapy is a common and important method to battle against ischemic stroke. The major mechanism of thrombolytic therapy is that profibrolysin is dissociated with plasmin, which can be used to degrade fibrin to dissolve thrombus. Unluckily, its application is limited and is only applied within 4.5–6 hours after suffering from it. Even if patients could be saved in time via thrombolytic therapy, there were only one-third to half of them accomplishing ischemic reperfusion [9], which might probably lead to transfer hemorrhagic stroke as a side effect.

Traditional Chinese medicine is always used without side effect to reduce disability rates, improve the quality of life, and decrease the risk of recrudesce for cerebral ischemic patients. The major mechanism involved anti-inflammatory reaction, antioxidant, inhibiting neuron apoptosis, promoting vascular regeneration, and maintaining the BBB permeability. AT was firstly recorded in *Sheng Nong's herbal classic* thousands years ago, and its dried rhizomes attributed to *Araceae Juss*, with the effect of reducing phlegm by resuscitation, improving intelligence, dehumidifying, and appetizing [10], which was used to treat tinnitus and epicophosis, Alzheimer disease, epilepsy, depression, and stroke. AT is one important component of Ditan decoction, widely used in clinical stroke treatment, which could decrease the level of inflammatory factors and oxidative stress after cerebral ischemic and inhibit the formation of thrombus to promote stroke patients recovery [11]. Many studies found that AT and its main ingredients had a positive effect on reducing the cerebral ischemic volume [12, 13], which was related to antioxidants [14], and regulating autophagy level [13, 15].

It is unclear whether AT and AT-contained ingredients have other mechanisms against IS. Due to lack of systematic and comprehensive explanation about it, network pharmacology was used to elucidate the interactive relation among drug, targets, and disease. Potential target prediction helps us to understand more mechanisms for multiple pharmacological functions of AT and its role in biological network, which will be beneficial for the curative effect improvement [16, 17]. Many kinds of complex compounds are involved in Chinese medicine, so we applied the network pharmacology method to reveal the effect of TCM. Thus, the aim of this paper is to find out the mechanism of AT against IS in a comprehensive and systemic way.

## 2. Methods and Materials

**Figure 1:**
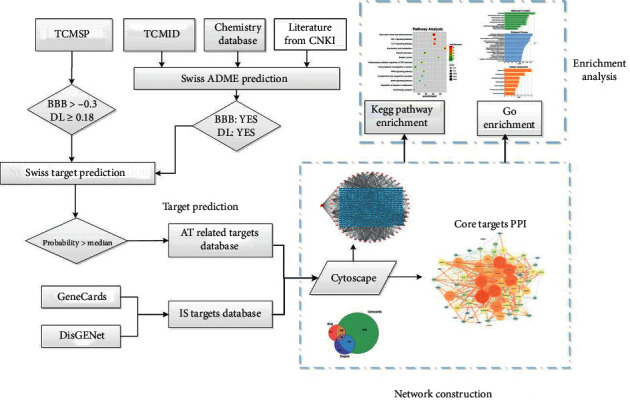
Framework for AT against IS based on a network pharmacology.

The flowchart of this study is shown in Figure 1.

### 2.1. Active Compound Screening and Target Prediction

We searched active compounds AT contained via TCMSP Database [18] (version2.3, http://tcmspw.com/tcmsp.php), TCMID Database (v2.01, http://119.3.41.228:8000/tcmid/search/), Chemistry Database [19] (http://chemdb.sgst.cn/scdb/default.asp), and literature from CNKI to provide more useful and important compounds. Based on pharmacokinetics (absorbed, distributed, metabolized, and excreted), we set up a series of parameters, such as BBB absorption>−0.3 and DL ≥ 0.18 [20], to screen suitable compounds in TCMSP Database. Compounds could be obtained from TCMID Database and Chemistry Database without ADME parameters, so we need to get chemical formula of them from PubChem (https://pubchem.ncbi.nlm.nih.gov/) to finish Swiss ADME prediction [21], which was requested that BBB permeability was equal to YES and at least two terms of Drug-likeness were yes. All compounds were screened as above-needed target prediction through Swiss Target [22]. Probability is used to balance the connection between compounds and targets, which is more close to 1, and it is more connective. We screened targets by a median of probability to establish potential target database related to compounds in AT.

### 2.2. Obtaining Gene Targets Related to IS

We obtained IS-related gene targets by searching in Genecards (version 4.14, https://www.genecards.org/) and DisGeNET [23–25] (version 7.0, https://www.disgenet.org/) with the keywords “ischemic stroke,” “cerebral ischemic,” “cerebral infarction.” These gene targets were put into IS-related targets database, respectively. Genes from Genecards in searching different keywords will be screened by its scores, which more than its median are constituted in IS-related targets database from Genecards.

### 2.3. AT-Compounds-Targets Network Construction

AT-compounds-targets visualized network was conducted by Cytoscape (version 3.7.2), where diversity of shape nodes was distinguished from AT, active compounds in AT, and targets, where edges represented the interactive relationship between AT and compounds and compounds and targets.

### 2.4. Extracting Core Target-Related AT-IS

By the aid of Microsoft Excel, we intersected potential target database related to compounds in AT- and IS-related target database to get the core target related to AT-IS, which was used to draw a Venn diagram online (http://www.bioinformatics.com.cn/).

### 2.5. Core Targets PPI Network Construction

Core targets related to AT-IS were imported into STRING (version 11.0, https://string-db.org/), selecting species “Homo sapiens”. Potential protein targets with a medium confidence score of 0.400 were designed as an interaction network, with hiding disconnect core targets to get PPI analysis results. All these results could be used in constructing AT-IS PPI visualized network, based on its degree value by analysing.

### 2.6. GO and KEGG Enrichment Analysis

GO and KEGG enrichment analysis of core targets related to AT-IS was accomplished by an online tool, Metascape [26] (https://metascape.org/), choosing species “Homo sapiens” and then clicking “custom analyse.” GO enrichment analysis is composed of biological process, molecular function, and cellular components. KEGG enrichment analysis is used to uncover the signal pathway showing potential therapeutic effect of some diseases. Before GO and KEGG enrichment for above core genes, “Min Overlap = 3,” “*P* value cutoff = 0.01,” and “Min enrichment = 1.5” should be set for passway and process enrichment and “Min Network Size = 3” and “Max Network Size = 500” for protein-protein interaction enrichment.

## 3. Results

### 3.1. Active Compounds in AT

There were 53 related active compounds satisfying ADME conditions, when filtering out repeat ones. MolID, molecular name, and Pubchem CID of AT active compounds are shown in Table 1. Structure, BBB permeability, and DL of these compounds are filled in Supplementary 3.

### 3.2. Compound Target Prediction Results and Its Network Construction

**Figure 2:**
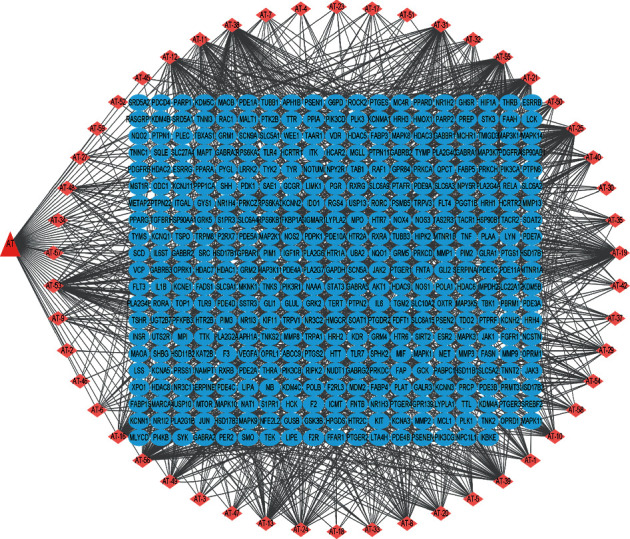
Drug-compounds-targets interaction network (there were 448 nodes and 1459 edges in this network, blue oval nodes represent genes, pink diamonds represent compounds contained in AT, and the red triangle represents AT).

Probability scores were used to predict these compound targets, which median is 0.0604024588 (AT-14 (palmitic acid) and AT-22 (2-furfuraldehyde) were lower than this median; no target was related to AT-28 blumenol A; thus, these three compounds were out of the network). 1409 targets were predicted, 398 targets remained after deleting repeat ones, and specific information is shown in Supplementary Table S1. Compound-target network is presented in Figure 2. The network property of compounds in AT, degree, betweenness centrality, closeness centrality, and neighborhood connectivity is involved in Table 2. Nodes with highest connectivity have a high influence on the network [29]. We proposed that the higher the degree it had, the more significant the effect it exerted in therapy. The top 10 degree compounds were bisasaricin, 3-cyclohexene-1-methanol-*α*,*α*,4-trimethyl,acetate, *cis*,*cis*,*cis*-7,10,13-hexadecatrienal, hydroxyacoronene, nerolidol, galgravin, veraguensin, 2′-o-methyl isoliquiritigenin, gamma-asarone, and alpha-asarone.

### 3.3. AT against IS Core Targets Protein-Protein Interaction (PPI) Construction

**Figure 3:**
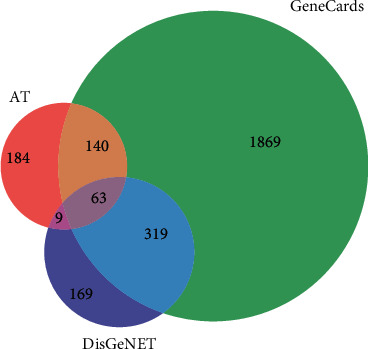
Venn diagram: intersection of genes between IS and AT. The part of three circles intersection represented the core target-related IS-AT.

We screened 2391 gene targets in Genecards Database, after filtering out based on median, when 560 gene targets were obtained from DisGeNET (Supplementary Table S2). Genecards Database was established based on gene-centric data from approximately 150 web sources, involved in genomic, transcriptomic, proteomic, genetic, clinical, and functional information. DisGeNET collected data from expert curated repositories, GWAS catalogues, animal models, and the scientific literature, which were identified well in relationship with disease. To avoid data redundancy, genes from Genecards and DisGeNET were intersected to improve targets relativity. In sum, 63 core gene targets remained after intersection with AT (Figure 3).

**Figure 4:**
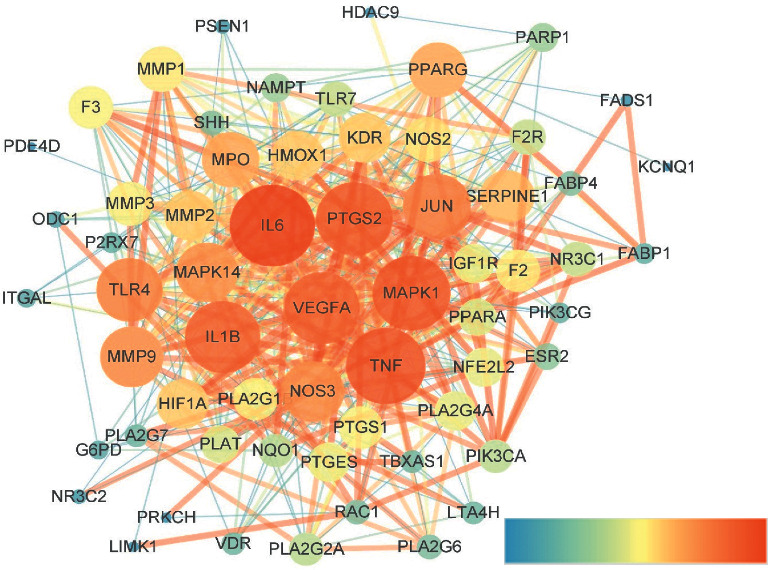
PPI network of potential core targets related to AT and IS. Left to right represented dark to light. The nodes in a lighter color and a bigger size represented a higher degree. The edges in a lighter color and a wider size represented a higher combined score.

Target GRM2 was dissociated from the PPI network, without interactive relationship, which should be omitted from it. Thus, 62 remaining targets are presented in Table 3, which were used to construct the PPI visualized network in Figure 4. The lighter color and the bigger size in nodes represented the higher degree value, which made a great contribution to its effect against IS.

### 3.4. GO Enrichment Analysis

**Figure 5:**
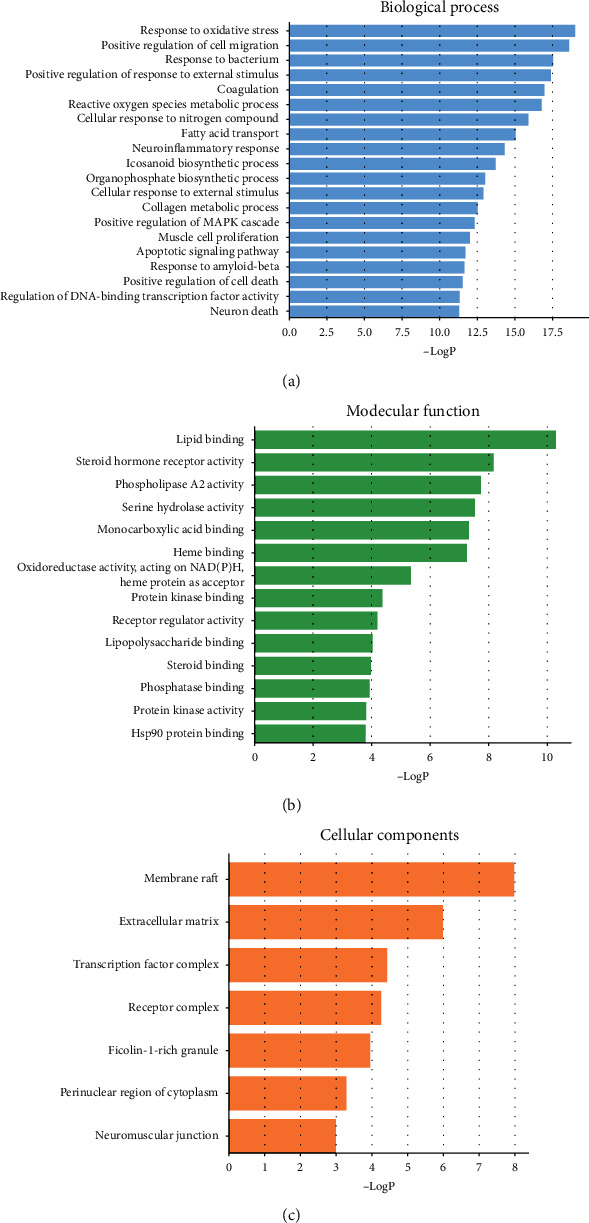
GO enrichment analysis: (a) the top 20 terms of biological process; (b) 14 terms of molecular function; (c) 7 terms of cell components with *P* < 0.05.

GO analysis of 62 potential core targets for AT against IS was performed by using the Metascape database to understand the relationship between functional units and their underlying significance in the biological system networks. The results were divided into three parts: biological processes (Figure 5(a)), cellular component (Figure 5(b)), and molecular function (Figure 5(c)), which were shown based on *P* < 0.05 in statistics.

Firstly, the top 20 terms of biological process for enrichment analysis are shown in Figure 5(a), such as response to oxidative stress, postive regulation of cell migration, response to bacterium, positive regulation of response to external stimulus, coagulation, reactive oxygen species metabolic process, cellular response to nitrogen compound, fatty acid transport, neuroinflammatory response, icosanoid biosynthetic process, organophosphate biosynthetic process, cellular response to external stimulus, collagen metabolic process, positive regulation of cell death, regulation of DNA-binding transcription factor activity, and neuron death. Secondly, there were only 7 terms of cellular components enrichment analysis shown in Figure 5(c) containing membrane raft, extracellular matrix, transcription factor complex, receptor complex, ficolin-1-rich granule, perinuclear region of cytoplasm, and neuromuscular junction. Finally, 14 terms of molecular function are presented in Figure 5(b), which were lipid binding, steroid hormone receptor activity, phospholipase A2 activity, serine hydrolase activity, monocarboxylic acid binding, heme binding, oxidoreductase activity acting on NADPH, protein kinase binding, receptor regulator activity, lipopolysaccharide binding, steroid binding, phosphatase binding, protein kinase activity, and hsp90 protein bind.

### 3.5. KEGG Enrichment Analysis

**Figure 6:**
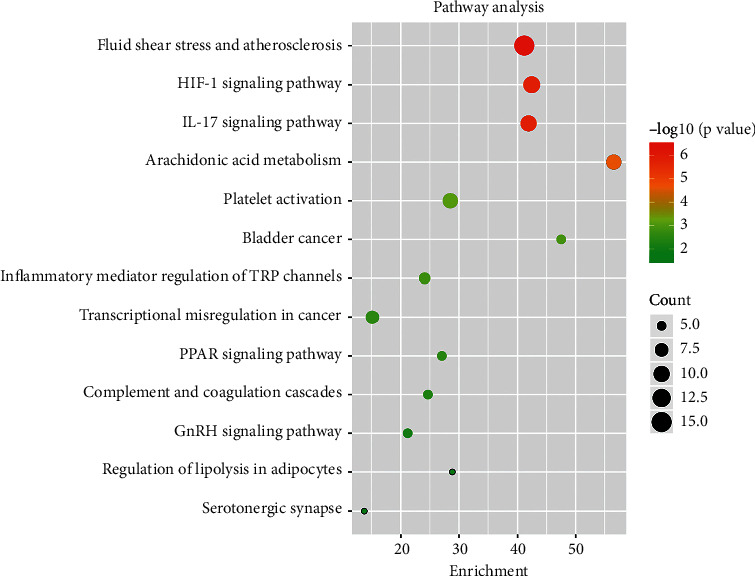
KEGG pathway enrichment analysis. “Enrichment” represented the number of target genes belonging to a pathway and the count of the annotated genes located in the pathway. The size of the dot represented the number of core genes related to AT-IS in the pathway and the color of the dot reflected the extent of significance in statistics (*P* < 0.05). Bigger size and lighter color of dot meant a higher level of enrichment.

**Figure 7:**
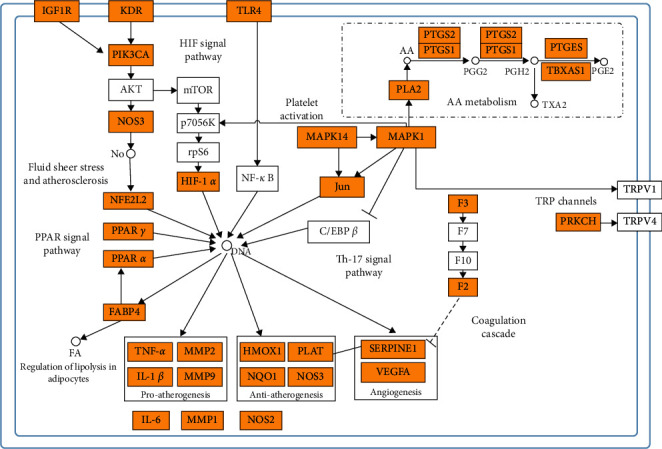
Systematic understanding of the antistroke mechanism of AT. The orange nodes represented the targets related to AT and IS and white ones related stroke. AA: arachidonic acid; FA: fatty acid. Full lines represent targets interacting with each other directly, and dotted lines represent indirect interaction.

**Figure 8:**
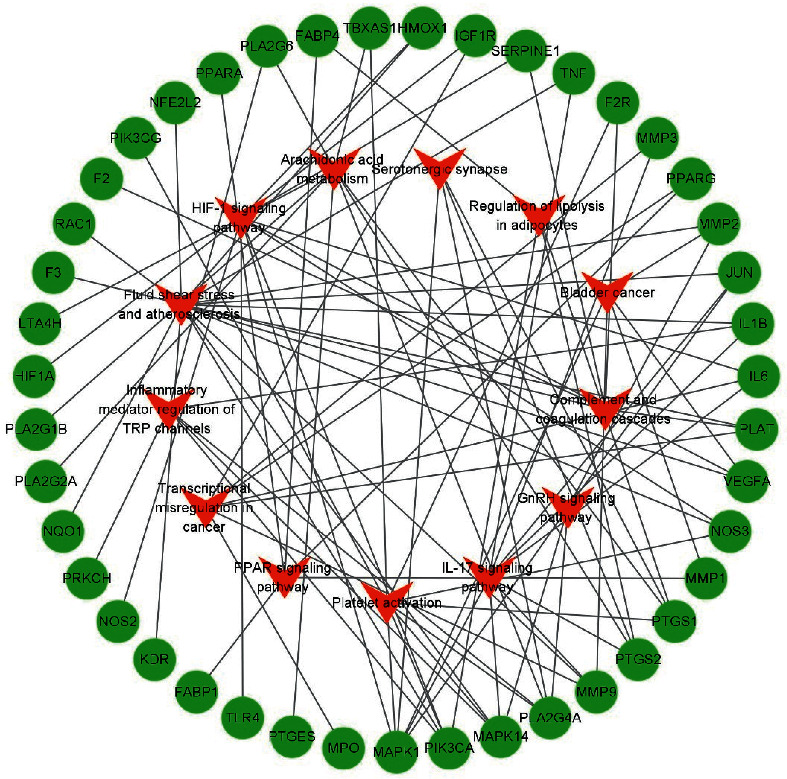
The network of potential core targets and signaling pathways in the treatment of IS by AT. Red nodes represent the potential signal pathway for AT against IS, and green nodes represent the potential core targets on it.

There were 13 terms of signal pathway of AT against IS by KEGG enrichment analysis, which were ordered as ascending tendency according to *P*-value(*P* < 0.05) and contained fluid shear stress and atherosclerosis, HIF-1 signaling pathway, IL-17 signaling pathway, arachidonic acid metabolism, platelet activation, bladder cancer, inflammatory mediator regulation of TRP channels, transcriptional misregulation in cancer, PPAR signaling pathway, complement and coagulation cascades, GnRH signaling pathway, regulation of lipolysis in adipocytes, and serotonergic synapse (Figure 6). According to KEGG enrichment analysis results, we made a systematic summary for these core targets in the signal pathway in Figure 7. And we were informed for the specific role of these targets in a certain signal pathway (Figure 8).

## 4. Discussion

Absorbed, distributed, metabolized, and excreted are significant parameters to determine the transformation and delivery of one drug in the body. A CNS-active drug having a high BBB permeability is crucial for its antistoke effect for entering into the brain. It was evidenced than if BBB permeability was lower than −0.30, and it had no effect on entering the brain through BBB, so we set it for more than that [20]. Only 6 compounds (bisasaricin, veraguensin, lupeol, cycloartenol, asatone, and eudesmin) fitted this ADME screening in TCMSP. So it was hard to point out AT in treating IS systemically and comprehensively, and we searched on TCMID Database, Chemistry Database, and literature to supply its composition to find more potential targets. In total, we found 50 compounds in AT with effect of treating IS probably, which mainly composed of volatile oil. *α*-Asarone and *β*-asarone accounted for 95% in volatile oil, both of which could promote neuron differentiation via PI3K by aid of growth factors [30]. In our study, it was confirmed that *α*-asarone, with a high degree value in compound-target network (degree = 59), uncovered its core role in AT.

There were 62 core targets for AT against IS, IL6, TNF, IL1B, TLR4, NOS3, MAPK1, PTGS2, VEGFA, JUN, and MMP9 involved in the top 10 degree targets. IL6, TNF, IL1B, TLR4, and NOS3 are related to the HIF-1 signal pathway, fluid shear stress and atherosclerosis, IL-17 signal pathway, and inflammatory mediator regulation of TRP channels, which are called inflammatory factors as effect for enhancing the level of inflammatory reaction in cerebral ischemic [31–33]. GO enrichment results contained some biological processes related to antistroke closely, such as response to oxidative stress, positive regulation of cell migration, coagulation, reactive oxygen species metabolic process, cellular response to nitrogen compound, fatty acid transport, and neuroinflammatory response. Similarly, some of KEGG enrichment pathways were related to secondary prevention of ischemic stroke by intervening atherosclerosis, regulation of lipolysis in adipocytes, coagulation cascades, arachidonic acid (AA) metabolism, and platelet activation. Others might have a potential therapeutic effect for IS, which were PPAR signal pathway, HIF-1 signal pathway, and inflammatory mediator regulation of TRP channels.

Atherosclerosis is the main pathological mechanism leading to cerebral ischemic, half of which has a connection with it. It was commonly known that it was beneficial to decrease the incidence or palindromia of cerebral ischemic by intervening atherosclerosis as secondary prevention [34]. In fact, fluid shear stress and atherosclerosis is a signal pathway with dual regulation for the formation of atherosclerosis, containing the biological process, antiatherosclerosis [35], and proatherosclerosis [36, 37], where nuclear transcripts Nrf2 and JUN (a part of AP-1 protein dimers) [38] are responsible for the process, respectively [39]. There was a evidence that lupeol, one component of AT, decreased lipid peroxidation level in the early stage of hypercholesterolemia artery atherosclerosis [40]. It was demonstrated that lupeol could exert a protective effect against cerebral I/R by activating Nrf2 and inhibiting p38-MAPK, with decreasing proinflammatory factors TNF-*α*, IL-1*β*, and IL-6, increasing anti-inflammatory factor IL-10, and suppressing oxidative stress level [41]. JNK and p38 MAPK promotes the expression of JUN to promote the proatherosclerosis process. Phosphorylation of p38-MAPK and JNK was inhibited by lupeol, when it suppresses the activation of microglia and astrocytes induced by LPS [42]. In addition, another component, bornyl acetate, which was found to promote HUVEC cell vitality recovery, insulted by ox-LDL, suppressing monocytes adhered to HUVEC cells, and decreasing TNF-*α* and IL-1*β* proinflammatory factors expression to antiatherosclerosis [43].

Disorder of fatty metabolism not only induces atherosclerosis but also is a high risk of stroke. It is necessary to regulate adipose lipolysis to avoid metabolism syndrome occurrence as possible to decrease the incidence of stroke indirectly. However, it required to further prove the role of adipose lipolysis in ischemic stroke for fat mice. It was confirmed that stroke rats with a high level of inflammatory factor in plasma and adipose tissue induced adipose lipolytic enzymes and free fatty acids expression increased [44]. It is a pity that amounts of compounds were conformed having a positive effect on fatty metabolism not through FABP4. Eudesmin could downregulate S6K1–H2BS36p to impair lipoblast differentiation [45]. Eugenol inhibited hepatic lipid accumulation by downregulating SREBP1 gene expression via increasing CAMKK, AMPK, and acetyl-CoA carboxylase (ACC) and suppressing phosphorylation of mammalian target of rapamycin (mTOR) and p70S6K [46]. Thymol could enhance PPAR*γ* and PPAR*δ* expression through overactivated p38MAPK, AMPK, and PKA to promote white adipose cell browning and increased lipid degeneration [47].

PPAR signal pathway has a close relationship with fatty metabolism, which also exerts a protective effect on cerebral ischemic. PPAR*α* and PPAR*γ*, nuclear transcription from nuclear receptor family, and PPAR*α*/*γ* agonist decreased inflammatory reaction to induce the neuroprotective effect after cerebral ischemic [48]. *α*-Asarone conjugate structure activated by PPAR*α*, increasing CPT1A gene expression related to degeneration and metabolism of fatty acid [49]. Linalool was identified as a PPAR*α* ligand directly [50], which improved postischemic neurological scores and cognitive ability by decreasing the COX-2, IL-6, Nrf expression in cortex, and hippocampus of IS rat [51].

Antiplatelet aggregative activity and anticoagulation are common and major therapy to prevent emboli and thrombus formation for ischemic stroke. Previous studies confirmed AT suppressing platelet activation and thrombus formation [52, 53]. Platelet activation and accumulation is a cascade process, and TXA_2_ is a positive feedback protein, platelet agonist, a second wave mediater, which is not unnecessary for its accumulation. It can trigger platelet activated by G protein-coupled receptor [54]. In our study, we found MAPK, ERK, PLA, PTGS1, and TBXAS1 core targets involved in the process of platelet activation to regulate TXA_2_ produce. SQ29548, TXA_2_R antagonist, was found to inhibit microglia/macrophages activation and enrichment to reduce injury of cerebral ischemic [55]. It has been proved based on network pharmacology that *β*-asarone could be against coagulation via APP, PTGS2, and TBXAS1 [56]. Eugenol inhibited platelet activation better than elemicin, in a dose-dependent manner, which also preceded the effect of ASA-COX inhibitors such as asprin [57]. Paeonol increased NO and PGI_2_ expression and decreased ET-1 and TXA_2_ expression to inhibit platelet activation and accumulation to suppress thrombus formation [58]. 3,11-Eudesmadien-2-one [59] and *α*-asarone [53] also had a similar effect, which is indispensable to further identify its specific mechanism.

In addition, arachidonic acid (AA) metabolism promotes platelet accumulation. COX-2(PTGS2) makes AA degeneration, whereas PLA2 promotes AA synthesis, which takes participation in TXA_2_ formation leading to platelet accumulation. A series of evidence has proved that a high level of COX-2 and PLA2 was filled with MCAO animal models and stroke patients [60–63]. Eugenol inhibited AA metabolism via cyclooxygenase and lipoxygenase pathways in human platelets [64]. In vitro, paeonol inhibited MAPKs activation in macrophages, decreasing iNOS, COX2, and IL6 expression to inhibit inflammatory reaction [65]. *α*-Iso-cubebene inhibited iNOS, COX2, and MMP9 expression and the phosphorylation of JNK and p38 to exert a neuroprotective effect, which was released by microglia amyloid beta induced [66]. However, whether paeonol or *α*-iso-cubebene has a neuroprotective effect for IS via inhibiting AA metabolism need to be further proved.

AT antithrombus was involved in an intrinsic way of coagulation cascades. FXa activated JNK pathway through PAR-1, which triggered F2 to induce neuron death [67]. In vitro, hippocampus clips insulted by OGD strengthened the activity of thrombus, which enhanced strength of synapsis by NAMDR; oppositely, if is inhibited thrombin/PAR-1, plasticity of synapsis was recovered [68]. It was a pity that there was no study proving AT or its components against thrombus by an intrinsic way of coagulation cascades so that we inferred it would be a novel research direction for neuroprotection of AT. Because of increasing Th-17 and decreasing Treg contributing to the pathology process of cerebral ischemic in clinic [69], we inferred that another new therapeutic point of view might be related to Th17 differentiation. In vivo, thymol decreased Th1/Treg and Th17/Treg in spleen for mice immune to Ova, which avoided overactivation of Th1 and Th17 [70].

Acute cerebral ischemic patients had a high level of HIF-1*α* in the early stage of clinic, indicating a serious expectation for 90 days [71]. TLR4 is one target in the HIF-1 signal pathway, where neutralizing HIF-1*α* weakened the increase in TLR4 in BV-2 cells in hypoxia condition with downregulation of TNF-*α* expression [72]. In fact, paeonol inhibited TLR4 expression, without influence of TNF-*α* [73]. *β*-Asarone had the ability of antioxidation, which decreased HIF-1*α* in cortex [74].

Both HRPA1 and TPR1 are members of TRP superfamily, which are structurally dependent nonselective cation channels and mediators of several signaling pathways. Methyl eugenol was proved to be hTPR1 agonist, selectively activating hTPRA1 [75]. Microglia was filled with TPRV1, which was activated to strengthen transmission of glutamate [76]. Previous study was identified that thymol improved spontaneous excitability transmission of spinal substantia gelatinosa neurons [77]. However, it is unknown whether methyl eugenol or thymol has a protective effect for IS via inflammatory mediator regulation of TRP channels, which needs more experiments to be proved. TPRV4 activated prompted expression of VEGFA and eNOS in cerebral ischemic mice, which was beneficial for proliferation and migration of neural stem cells (NPCs) and angiogenesis [78]. TPRV4 agonist impaired cerebral in IS rats by upregulating PI3K/AKT and downregulating p38MAPK (MAPK14) [79].

Taken together, these signal pathways, related to AT preventing and treating IS, could be approximately classified into three categories as follows: atherosclerosis, regulation of lipolysis in adipocytes, and PPAR signal pathway were associated with lipid metabolism; coagulation cascades, arachidonic acid (AA) metabolism, and platelet activation had a connection with the therapy of antiplatelet aggregative activity and anticoagulation; and HIF-1 signal pathway, inflammatory mediator regulation of TRP channels, and Th17 differentiation might be the potential therapeutic signal pathway. In addition, we inferred that an intrinsic way of coagulation cascades and Th17 differentiation was probably new therapeutic or preventive direction for the role of AT against IS.

## 5. Conclusion

In summary, we explored and explained multiple compounds, multiple pathways, and multiple targets of AT-regulated ischemic stroke treatment based on a network pharmacology method. Our data indicated that amounts of compounds contained in AT might prevent and treat ischemic stroke through some signal pathways, most of which need an elaborated detailed mechanism in the related signal pathway. Two novel research directions (IL-17 signaling pathway and complement and coagulation cascades) of AT therapeutic for preventing IS were proposed. In the future, more studies should focus on providing experimental evidence and enhancing the effect of AT in the cerebral ischemic on a comprehensive level and improving ability of AT targeting to the brain and crossing the BBB.

## Figures and Tables

**Table 1:** Compounds contained in AT.

No.	MolID	Molecular name	PubChem CID	Resource
1	AT-1	Linalool	6549	[27]
2	AT-2	(+)-Longicyclene	564934	[27]
3	AT-3	(±)-Camphor	159055	[27]
4	AT-4	Bornyl acetate	6448	[27]
5	AT-5	(−)-Terpinen-4-ol	5325830	[27]
6	AT-6	Borneol	64685	[27]
7	AT-7	Shyobunone	5321293	[27]
8	AT-8	Methyl eugenol	7127	[27]
9	AT-9	(+)-Ledol	92812	[27]
10	AT-10	(E)-Methyl isoeugenol	1549045	[27]
11	AT-11	Elemicin	10248	[27]
12	AT-12	Alpha-asarone	636822	[27]
13	AT-13	*β*-Asarone	5281758	[27]
14	AT-14	Palmitic acid	985	[27]
15	AT-16	Galgravin	101749	[28]
16	AT-17	(7S,7′S,8R,8′R)-Eudesmin	284823	[28]
17	AT-18	1,1,7-Trimethyl-4-methene-decahydro-1H-cycloprop[e]azulene	91753508	TCMID
18	AT-19	Gamma-asarone	636750	TCMID
19	AT-20	1H-Cyclopropa [a] naphthalene,1a,2,3,5,6,7,7a,7b-octahydro-1,1,7,7a-tetramethyl,[1aR-(1a. Alpha., 7. Alpha., 7b. Alpha.)]	71717616	TCMID
20	AT-21	2′-o-Methyl isoliquiritigenin	5319688	TCMID
21	AT-22	2-Furfuraldehyde	7362	TCMID
22	AT-23	Beta-eudesmol-*cis*-epimer	12309818	TCMID
23	AT-24	3,11-Eudesmadien-2-one	565648	TCMID
24	AT-25	3-Cyclohexene-1-methanol-*α*,*α*,4-trimethyl,acetate	11736872	TCMID
25	AT-27	Asaronaldehyde	20525	TCMID
26	AT-28	Blumenol A	5280462	TCMID
27	AT-29	Carotol	6432448	TCMID
28	AT-30	Caryophyllene epoxid	14350	TCMID
29	AT-31	*cis*,*cis*,*cis*-7,10,13-Hexadecatrienal	5367366	TCMID
30	AT-32	*cis*-1′,2′-Epoxyasarone	101009521	TCMID
31	AT-33	Endo-borneol	1201518	TCMID
32	AT-34	Paeonol	11092	TCMID
33	AT-35	Eugenol	3314	TCMID
34	AT-37	Himbaccol	6432543	TCMID
35	AT-38	Hydroxyacoronene	102585857	TCMID
36	AT-39	Isoacoramone	700861	TCMID
37	AT-40	Isolongifolen-5-one	600416	TCMID
38	AT-42	Naphthalene,1,2,3,4-tetrahydro-1,5,7-trimethyl	529436	TCMID
39	AT-45	Octenol	5352836	TCMID
40	AT-46	Phenol,2-methoxy	460	TCMID
41	AT-47	Terpinen-4-ol-	20398672	TCMID
42	AT-48	Thymol	6989	TCMID
43	AT-49	Methylisoeugenol	637776	[19]
44	AT-50	Graminone	36679	[19]
45	AT-51	*α*-Cubebene	442359	[19]
46	AT-52	*β*-Cubebene	93081	[19]
47	AT-53	Nerolidol	5284507	[19]
48	AT-54	Guaiol	227829	[19]
49	AT-55	Bisasaricin	126324	[19]
50	AT-56	Veraguensin	443026	[18]
51	AT-57	Lupeol	259846	[18]
52	AT-58	Cycloartenol	92110	[18]
53	AT-59	Asatone	10983193	[18]

**Table 2:** Property of compounds in AT.

No.	Molid	Betweenness centrality	Closeness centrality	Degree	Neighborhood connectivity
1	AT-55	0.132711	0.389373	80	5.319444
2	AT-25	0.128075	0.393486	78	6.102564
3	AT-31	0.125837	0.390734	74	5.716216
4	AT-38	0.093817	0.390052	73	7.191781
5	AT-53	0.097086	0.384682	67	6.030769
6	AT-16	0.071401	0.382705	66	6.322581
7	AT-56	0.07275	0.382705	66	6.225806
8	AT-21	0.141358	0.384021	64	4.390625
9	AT-19	0.044583	0.378173	61	8.690909
10	AT-12	0.041561	0.376897	59	8.471698
11	AT-39	0.098263	0.378173	55	5.690909
12	AT-11	0.04764	0.373746	48	8.3125
13	AT-13	0.03325	0.373122	47	8.978723
14	AT-24	0.023989	0.370647	43	9.72093
15	AT-49	0.02007	0.3646	39	10.09091
16	AT-8	0.015872	0.364007	36	10.28125
17	AT-10	0.029031	0.365794	35	9.428571
18	AT-20	0.013698	0.362237	29	9.827586
19	AT-40	0.01066	0.36165	28	11.21429
20	AT-1	0.023586	0.361066	27	8.222222
21	AT-29	0.014423	0.361066	27	8.703704
22	AT-54	0.013264	0.360484	26	10.46154
23	AT-35	0.038727	0.3576	21	7.095238
24	AT-33	0.009883	0.357029	20	11.65
25	AT-5	0.009883	0.357029	20	11.65
26	AT-48	0.018746	0.355326	19	10.64706
27	AT-50	0.029511	0.356459	19	8.052632
28	AT-32	0.013807	0.355326	17	11.11765
29	AT-47	0.018568	0.354762	16	10.5
30	AT-58	0.013403	0.354762	16	13.3125
31	AT-17	0.005603	0.3542	15	10
32	AT-6	0.007742	0.3542	15	12.73333
33	AT-7	0.007742	0.3542	15	12.73333
34	AT-57	0.007296	0.353639	14	13.57143
35	AT-3	0.003614	0.353081	13	12.23077
36	AT-34	0.019332	0.353081	13	8.076923
37	AT-37	0.007211	0.352524	12	14.33333
38	AT-9	0.003189	0.352524	12	14.83333
39	AT-42	0.015185	0.351969	11	11.18182
40	AT-23	0.004943	0.351415	10	16.9
41	AT-27	0.027215	0.351415	10	7.5
42	AT-4	0.002483	0.350863	9	13.66667
43	AT-18	9.74E-04	0.349219	6	19.33333
44	AT-30	0.004804	0.348674	5	16.8
45	AT-46	0.001824	0.348674	5	15
46	AT-51	4.81E-04	0.348674	5	19.6
47	AT-45	3.60E-04	0.348131	4	18.75
48	AT-59	0.005322	0.348131	4	14.75
49	AT-2	2.51E-04	0.347589	3	24.66667
50	AT-52	2.32E-05	0.34705	2	37

**Table 3:** Potential targets related to IS and AT.

No.	Gene name	Protein name	UniProt ID	Degree
1	IL6	Interleukin-6	P05231	46
2	TNF	TNF-alpha	P01375	42
3	MAPK1	MAP kinase ERK2	P28482	41
4	PTGS2	Cyclooxygenase-2	P35354	40
5	IL1B	Interleukin-1 beta	P01584	39
6	VEGFA	Vascular endothelial growth factor A	P15692	39
7	JUN	Proto-oncogene c-JUN	P05412	34
8	TLR4	Toll-like receptor 4	O00206	33
9	MMP9	Matrix metalloproteinase 9	P14780	31
10	NOS3	Nitric-oxide synthase, endothelial	P29474	31
11	MAPK14	MAP kinase p38 alpha	Q16539	30
12	MPO	Myeloperoxidase	P05164	27
13	PPARG	Peroxisome proliferator-activated receptor gamma	P37231	27
14	SERPINE1	Plasminogen activator inhibitor-1	P05121	25
15	MMP2	Matrix metalloproteinase 2	P08253	23
16	HIF1A	Hypoxia-inducible factor 1 alpha	Q16665	23
17	HMOX1	HMOX1	P09601	23
18	KDR	Vascular endothelial growth factor receptor 2	P35968	23
19	MMP1	Matrix metalloproteinase 1	P03956	20
20	NOS2	Nitric oxide synthase, inducible	P35228	20
21	MMP3	Matrix metalloproteinase 3	P08254	19
22	PTGS1	Cyclooxygenase-1	P23219	18
23	PLA2G1B	Phospholipase A2 group 1B	P04054	18
24	F3	Coagulation factor VII/tissue factor	P13726	18
25	F2	Thrombin	P00734	18
26	PLA2G4A	Cytosolic phospholipase A2	P47712	16
27	PTGES	Prostaglandin E synthase	O14684	16
28	IGF1R	Insulin-like growth factor I receptor	P08069	16
29	NFE2L2	Nuclear factor erythroid 2-related factor 2	Q16236	16
30	PPARA	Peroxisome proliferator-activated receptor alpha	Q07869	15
31	PLAT	Tissue-type plasminogen activator	P00750	15
32	TLR7	Toll-like receptor (TLR7/TLR9)	Q9NYK1	14
33	NR3C1	Glucocorticoid receptor	P04150	14
34	F2R	Proteinase-activated receptor 1	P25116	14
35	PLA2G2A	Phospholipase A2 group IIA	P14555	13
36	NQO1	Quinone reductase 1	P15559	13
37	PIK3CA	PI3-Kinase p110-alpha subunit	P42336	13
38	PARP1	Poly[ADP-ribose]polymerase-1	P09874	11
39	NAMPT	Nicotinamide phosphoribosyltransferase	P43490	11
40	ESR2	Estrogen receptor beta	Q92731	10
41	SHH	Sonic hedgehog protein (by homology)	Q15465	9
42	PLA2G6	Calcium-independent phospholipase A2	O60733	9
43	RAC1	Ras-related C3 botulinum toxin substrate 1	P63000	9
44	FABP4	Fatty acid binding protein adipocyte	P15090	9
45	VDR	Vitamin D receptor	P11473	8
46	LTA4H	Leukotriene A4 hydrolase	P09960	8
47	TBXAS1	Thromboxane-A synthase	P24557	8
48	PLA2G7	LDL-associated phospholipase A2	Q13093	8
49	P2RX7	P2X purinoceptor 7	Q99572	7
50	PIK3CG	PI3-Kinase p110-gamma subunit	P48736	7
51	FABP1	Fatty acid-binding protein, liver (by homology)	P07148	7
52	G6PD	Glucose-6-phosphate 1-dehydrogenase	P11413	6
53	ITGAL	Leukocyte adhesion glycoprotein LFA-1 alpha	P20701	6
54	ODC1	Ornithine decarboxylase	P11926	5
55	PSEN1	Presenilin-1 (PS-1) (EC 3.4.23.-) (protein S182) [cleaved into presenilin-1 NTF subunit; presenilin-1 CTF subunit; presenilin-1 CTF12 (PS1-CTF12)]	P49768	4
56	NR3C2	Mineralocorticoid receptor	P08235	4
57	FADS1	Fatty acid desaturase 1	O60427	3
58	HDAC9	Histone deacetylase 9	Q9UKV0	2
59	PRKCH	Protein kinase C eta	P24723	2
60	LIMK1	LIM domain kinase 1	P53667	2
61	PDE4D	Phosphodiesterase 4D	Q08499	1
62	KCNQ1	Voltage-gated potassium channel, IKs; KCNQ1(Kv7.1)/KCNE1(MinK)	P51787	1

## Data Availability

The data used to support the findings of this study are available from the corresponding author upon request.

## Supplementary Materials

Supplementary Materials Table S1: compound targets for each component in AT by prediction. Table S2: IS-related targets in GeneCards and DisGeNET. Table S3: specific property of compounds contained in AT.
