# Supplementary material for: Revealing the Pharmacological Mechanism of Acorus tatarinowii in the Treatment of Ischemic Stroke Based on Network Pharmacology
Source: Evid Based Complement Alternat Med. 2020 Oct 31;2020:3236768. doi: 10.1155/2020/3236768 (PMC7648688; doi:10.1155/2020/3236768)
Supplement: Supplementary Materials — Table S1: compound targets for each component in AT by prediction. Table S2: IS-related targets in GeneCards and DisGeNET. Table S3: specific property of compounds contained in AT. [file 3236768.f1.zip › Supplementary3.docx]

| NO. | MOLID | Molecular Name | Pubchem CID | Structure | BBB(%) | DL |
| --- | --- | --- | --- | --- | --- | --- |
| 1 | AT-1 | Linalool | 6549 | 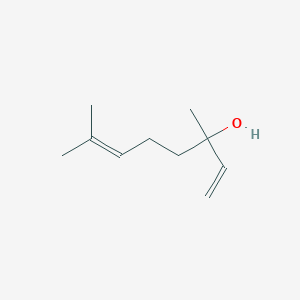 | YES | YES |
| 2 | AT-2 | (+)-longicyclene | 564934 | 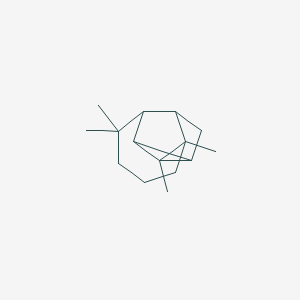 | YES | YES |
| 3 | AT-3 | (±)-Camphor | 159055 | 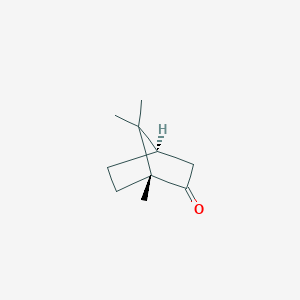 | YES | YES |
| 4 | AT-4 | Bornyl acetate | 6448 | 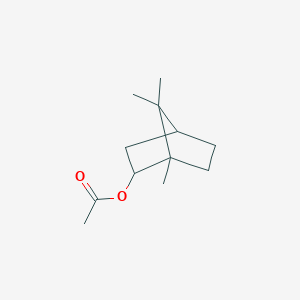 | YES | YES |
| 5 | AT-5 | (-)-Terpinen-4-ol | 5325830 | 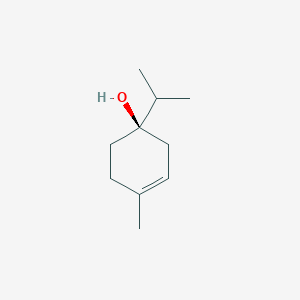 | YES | YES |
| 6 | AT-6 | borneol | 64685 | 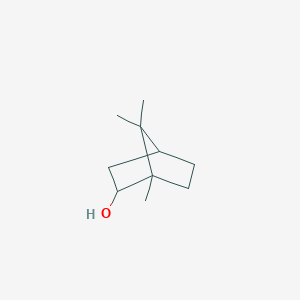 | YES | YES |
| 7 | AT-7 | shyobunone | 5321293 | 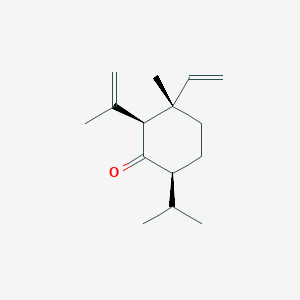 | YES | YES |
| 8 | AT-8 | Methyl Eugenol | 7127 | 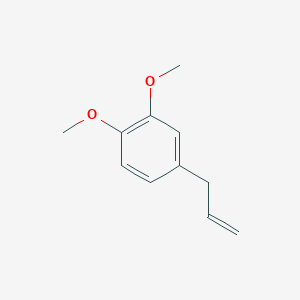 | YES | YES |
| 9 | AT-9 | (+)-ledol | 92812 | 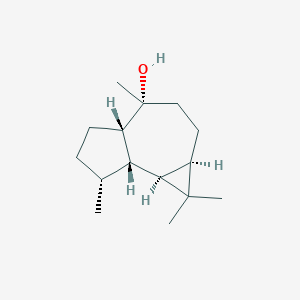 | YES | YES |
| 10 | AT-10 | (E)-methyl isoeugenol | 1549045 | 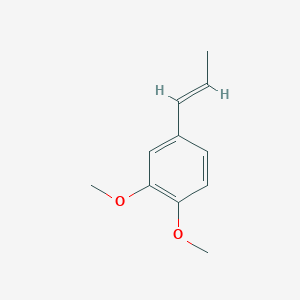 | YES | YES |
| 11 | AT-11 | Elemicin | 10248 | 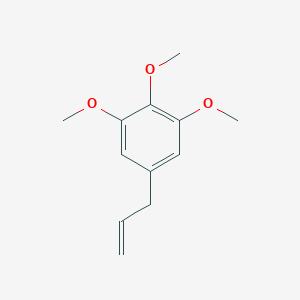 | YES | YES |
| 12 | AT-12 | alpha- Asarone | 636822 | 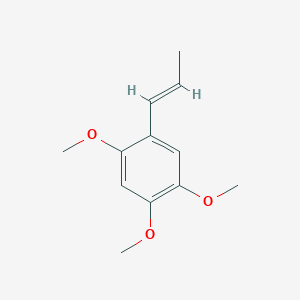 | YES | YES |
| 13 | AT-13 | β-asarone | 5281758 | 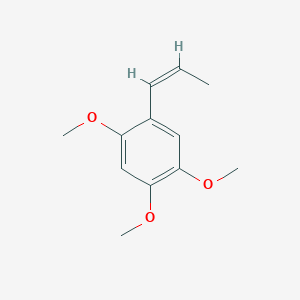 | YES | YES |
| 14 | AT-14 | Palmitic acid | 985 | 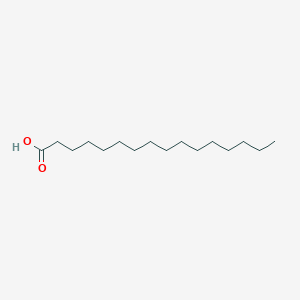 | YES | YES |
| 15 | AT-16 | galgravin | 101749 | 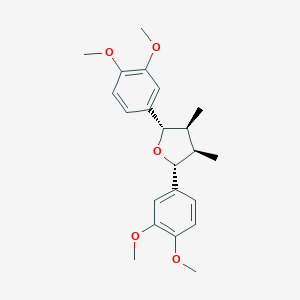 | YES | YES |
| 16 | AT-17 | (7S,7’S,8R,8’R)-eudesmin | 284823 | 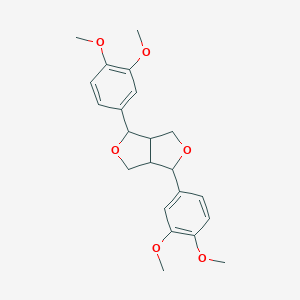 | YES | YES |
| 17 | AT-18 | 1,1,7-trimethyl-4-methene-decahydro-1H-cycloprop[e]azulene | 91753508 | 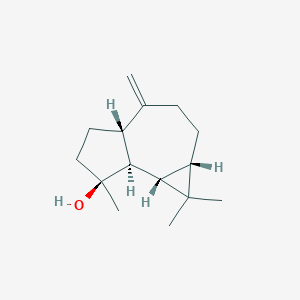 | YES | YES |
| 18 | AT-19 | gamma-Asarone | 636750 | 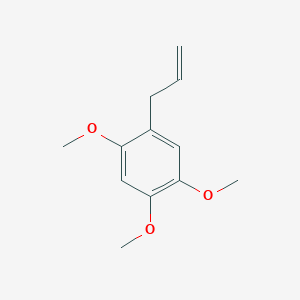 | YES | YES |
| 19 | AT-20 | 1H- Cyclopropa［a］naphthalene,1a,2,3,5,6,7,7a,7b- octahydro- 1,1,7,7a- tetramethyl-,［1aR-(1a． alpha． ,7． alpha． ,7b． alpha． )］ | 71717616 | 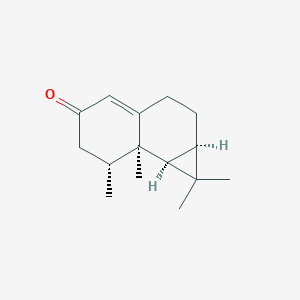 | YES | YES |
| 20 | AT-21 | 2'-o-methyl isoliquiritigenin | 5319688 | 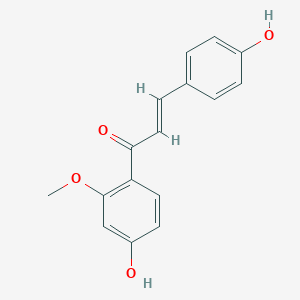 | YES | YES |
| 21 | AT-22 | 2- Furfuraldehyde | 7362 | 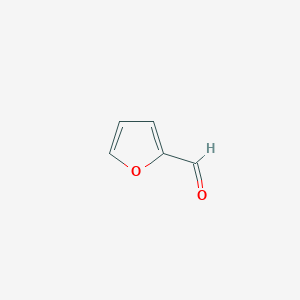 | YES | YES |
| 22 | AT-23 | beta-Eudesmol cis epimer | 12309818 | 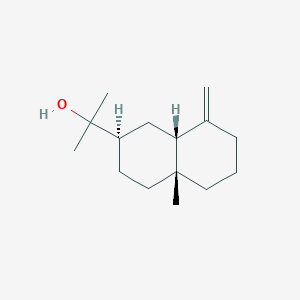 | YES | YES |
| 23 | AT-24 | 3,11-eudesmadien-2-one | 565648 | 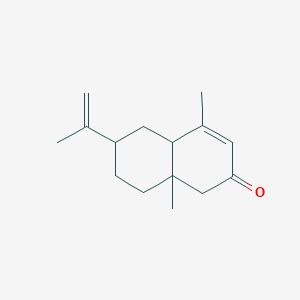 | YES | YES |
| 24 | AT-25 | 3-cyclohexene-1-methanol-α,α,4-trimethyl,acetate | 11736872 | 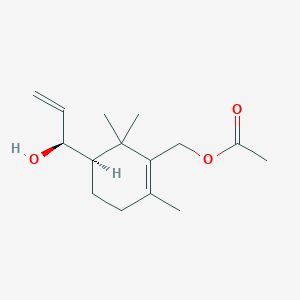 | YES | YES |
| 25 | AT-27 | asaronaldehyde | 20525 | 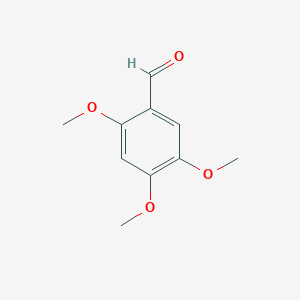 | YES | YES |
| 26 | AT-28 | blumenol a | 5280462 | 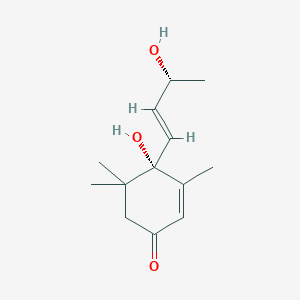 | YES | YES |
| 27 | AT-29 | carotol | 6432448 | 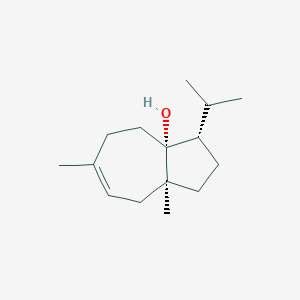 | YES | YES |
| 28 | AT-30 | caryophyllene epoxid | 14350 | 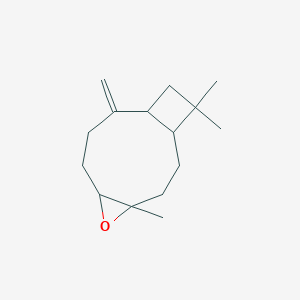 | YES | YES |
| 29 | AT-31 | cis,cis,cis-7,10,13-hexadecatrienal | 5367366 | 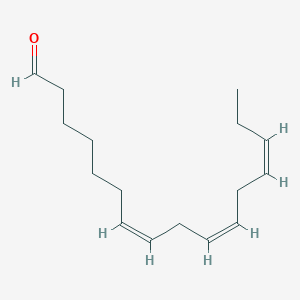 | YES | YES |
| 30 | AT-32 | cis-1',2'-epoxyasarone | 101009521 | 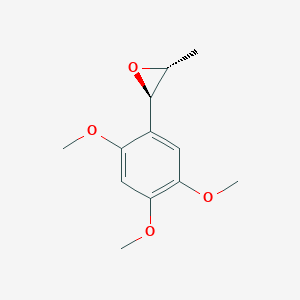 | YES | YES |
| 31 | AT-33 | endo- Borneol- | 1201518 | 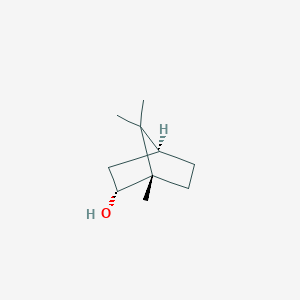 | YES | YES |
| 32 | AT-34 | PAEONOL | 11092 | 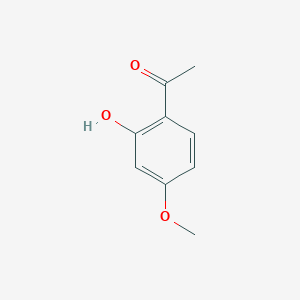 | YES | YES |
| 33 | AT-35 | eugenol | 3314 | 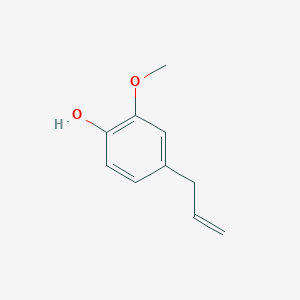 | YES | YES |
| 34 | AT-37 | himbaccol | 6432543 | 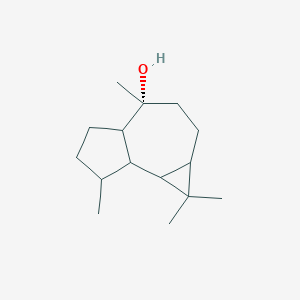 | YES | YES |
| 35 | AT-38 | hydroxyacoronene | 102585857 | 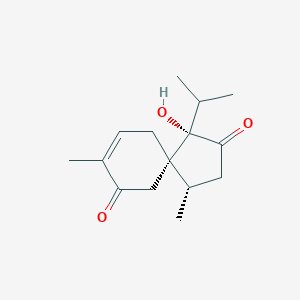 | YES | YES |
| 36 | AT-39 | isoacoramone | 700861 | 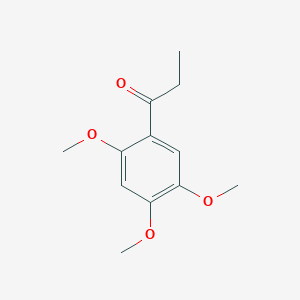 | YES | YES |
| 37 | AT-40 | isolongifolen-5-one | 600416 | 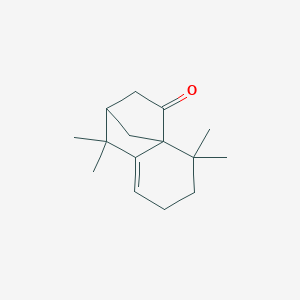 | YES | YES |
| 38 | AT-42 | Naphthalene,1,2,3,4- tetrahydro- 1,5,7- trimethyl | 529436 | 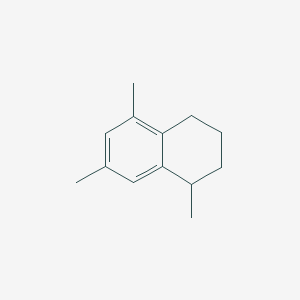 | YES | YES |
| 39 | AT-45 | octenol | 5352836 | 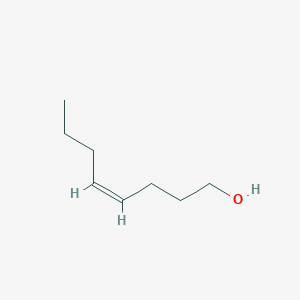 | YES | YES |
| 40 | AT-46 | phenol,2- methoxy | 460 | 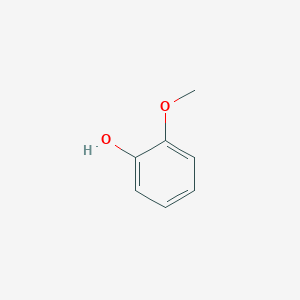 | YES | YES |
| 41 | AT-47 | terpinen- 4- ol- | 20398672 | 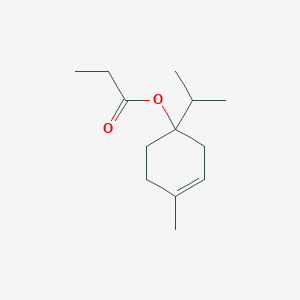 | YES | YES |
| 42 | AT-48 | thymol | 6989 | 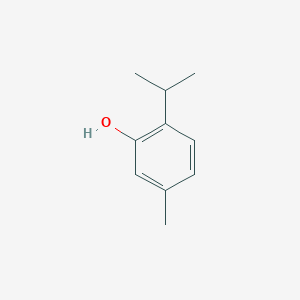 | YES | YES |
| 43 | AT-49 | methylisoeugenol | 637776 | 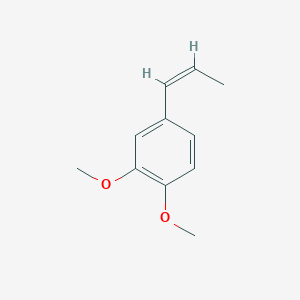 | YES | YES |
| 44 | AT-50 | Graminone | 36679 | 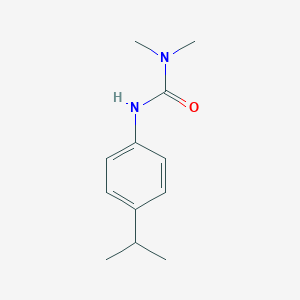 | YES | YES |
| 45 | AT-51 | α-cubebene | 442359 | 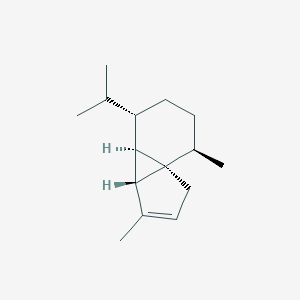 | YES | YES |
| 46 | AT-52 | β-cubebene | 93081 | 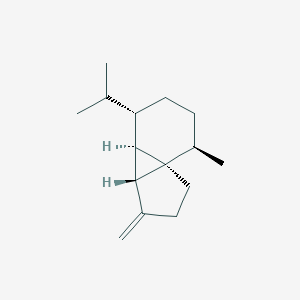 | YES | YES |
| 47 | AT-53 | nerolidol | 5284507 | 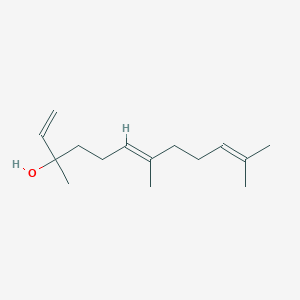 | YES | YES |
| 48 | AT-54 | guaiol | 227829 | 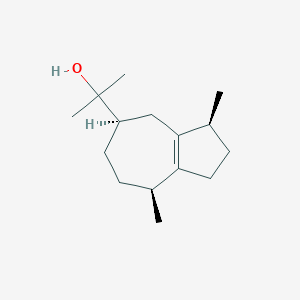 | YES | YES |
| 49 | AT-55 | bisasaricin | 126324 | 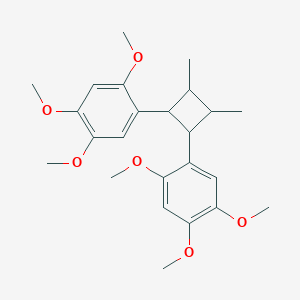 | 0.54 | 0.5 |
| 50 | AT-56 | Veraguensin | 443026 | 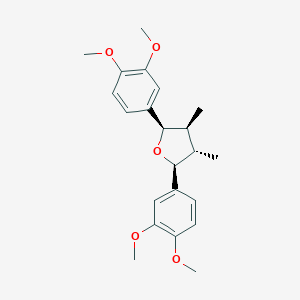 | 0.72 | 0.39 |
| 51 | AT-57 | lupeol | 259846 | 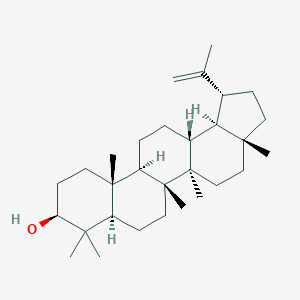 | 1.29 | 0.78 |
| 52 | AT-58 | Cycloartenol | 92110 | 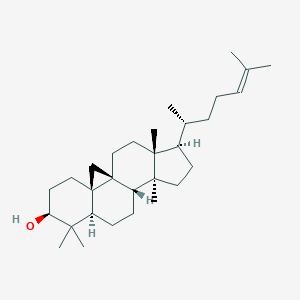 | 1.33 | 0.78 |
| 53 | AT-59 | Asatone | 10983193 | 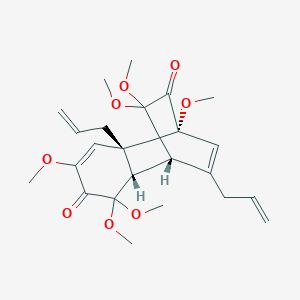 | 0.16 | 0.52 |
